# Mechanically induced development and maturation of 3D *in-vitro* organoid platform: an organotypic heterogeneous microphysiological model of patient-derived organoids with ER/PR/HER2^+^ breast cancer

**DOI:** 10.3389/fimmu.2025.1594405

**Published:** 2025-07-31

**Authors:** Mamta Kumari, Kamare Alam, Anamitra Bhattacharya, Nakka Sharmila Roy, Vaishnavi Madhasu, Bitan Guchhait, Sangita Dan, Soma Sett, Jayanta Chakrabarti, Chandan Mandal, Velayutham Ravichandiran, Subhadeep Roy

**Affiliations:** ^1^ Department of Pharmaceutics, National Institute of Pharmaceutical Education and Research, Kolkata, West Bengal, India; ^2^ Department of Pharmacology and Toxicology, National Institute of Pharmaceutical Education and Research, Kolkata, West Bengal, India; ^3^ Surgical Oncology, Chittaranjan National Cancer Institute, Kolkata, India; ^4^ Molecular Pathology, Department of Laboratory of Medicine, Chittaranjan National Cancer Institute, Kolkata, India; ^5^ Department of Natural Products, National Institute of Pharmaceutical Education and Research, Kolkata, West Bengal, India

**Keywords:** breast cancer, patient-derived organoid, 3D microphysiological model, tumor heterogeneity, extracellular matrix, personalized medicine

## Abstract

**Introduction:**

Breast cancer comprises diverse histological and molecular subtypes, each characterized by distinct biological behaviors and therapeutic responses. So, to unravel the biological complexity of cancer tissue, we must research it down to the heterogeneous cell level, where one can investigate and deconstruct the molecular and biochemical characteristics of various cell types (Fibroblast, Endothelial Cells, and Cancer Stem cells). Unfortunately, advancements have been hindered due to the absence of thorough methods for identifying, isolating, and cultivating all patient-derived organoids (PDOs) types from various tissues. Personalized therapy in the form of PDOs represents a promising approach to advance therapeutic outcomes. This study aims to replicate the cellular and molecular heterogeneity of breast cancer by examining multiple cell types within PDOs and their contributions to tumor progression and metastasis.

**Methods:**

We developed and characterized 3D in vitro PDO models from breast cancer tissues, encompassing various subtypes including ER+, PR+, and HER2+ tumors. We have sought to comprehend the fundamental nature of the various breast cancer cell types uncover the biology underlying their inherent characteristics, the outcomes of their interactions, and the contributions they provide to the metastatic potential. The IHC finding showed the positive expression for B cells (CD20), luminal epithelial cells (CD24), leukocytes infiltrating cells (CD45), mesenchymal stem cells (CD73, CD90, 105), vascular endothelial cells (CD34, CD105), EMT (E-cadherin), and fibroblast (Fibronectin, collagen, laminin) markers. In addition, we provide a new IHC/IF antibody panel and a stringent identification that can address significant breast cancer cells. Oxidative stress biomarkers and secretome analysis patterns were analyzed to identify the release pattern of variable pro-inflammatory growth cytokines produced by the endothelial and cancer stem cells.

**Results:**

The IHC finding showed the positive expression for B cells (CD20), luminal epithelial cells (CD24), leukocytes infiltrating cells (CD45), mesenchymal stem cells (CD73, CD90, 105), vascular endothelial cells (CD34, CD105), EMT (E-cadherin), and fibroblast (Fibronectin, collagen, laminin) markers. In addition, we provide a new IHC/IF antibody panel and a stringent identification that can address significant breast cancer cells. Oxidative stress biomarkers and secretome analysis patterns were analyzed to identify the release pattern of variable pro-inflammatory growth cytokines produced by the endothelial and cancer stem cells.

**Discussion:**

The findings revealed the diverse fibroblast heterogeneity and variable epithelial to molecular profiles consistent with the original breast tumor. These 3D *in vitro* PDO models are essential for investigating the complex cellular interactions in breast cancer cells. This collection of research provides a fresh look at the model and serves as a valuable tool for developing tailored treatment strategies and facilitating personalized therapeutic approaches for breast cancer patients by illuminating its biochemical, cellular, and molecular make-up.

## 1 Introduction

Breast cancer is the most frequently diagnosed cancer worldwide, affecting millions of women each year and classified into distinct subtypes based on the histopathological and molecular characteristics of the primary tumor. These subtypes include estrogen receptor-positive (ER+), progesterone receptor-positive (PR+), Human epidermal growth factor receptor-2-positive (HER2+), and triple-negative breast cancer (TNBC) (1). In 2020, there were an estimated 2.3 million new cases of breast cancer globally, making it a significant public health concern (2). Despite significant advancements in early detection through screenings, development of targeted therapies, and a deeper understanding of genetic predisposition, it remains the second leading cause of cancer-related mortality among females, reflecting profound disease heterogeneity and metastasis (3). By 2040, the incidence of breast cancer is expected to increase by 3 million new cases annually, with mortality rates reaching approximately 1 million per year (4). The onset and progression of breast cancer are influenced by various interconnected factors, including tumor microenvironment (TME), which plays a key role in regulating cancer pathogenesis, progression, metastasis, invasiveness, relapse, and therapy resistance (5). Additionally, inter- and intra-patient tumor heterogeneity presents a major challenge in achieving optimal therapeutic outcomes, as it often results in variable responses to anticancer treatments. Certain therapies may demonstrate efficacy in some patients while failing to produce favorable results in others (6). The breast tumor microenvironment is complex and dynamic, comprising a heterogeneous population of cells, including cancer-associated fibroblasts (CAFs), endothelial cells (ECs), epithelial cells, mesenchymal stem cells (MSCs), immune cells, angiogenic cells, and an altered extracellular matrix (ECM) (7, 8). This intricate network is crucial in cancer progression, metastasis, and therapy resistance. CAFs, as the most abundant stromal component, actively interact with tumor cells and other stromal elements, facilitating tumor growth and invasion through ECM remodeling and modulation of immune responses (9). The ECM serves as a structural scaffold and influences cellular behavior, with its composition and organization being critical in cancer development. Additionally, the interplay between CAFs and immune cells within the TME significantly impacts the progression of cancer and treatment response (10). Despite advancements in understanding the pathobiological complexity of breast cancer, there is a continued need for novel molecular and pharmacogenomic markers to predict drug responses accurately. Traditional two-dimensional (2D) *in vitro* models and animal models, present notable limitations when it comes to accurately mimicking the multifaceted nature of human breast tumors. In contrast, three-dimensional (3D) models are invaluable tools in cancer research, as they closely mimic the *in vivo* tumor architecture and microenvironment, enabling more accurate predictions of drug responses (11). Among these models, organoids have garnered significant attention for their ability to emulate tumor biology’s critical features. More recently, patient-derived organoids (PDOs) have emerged as a promising platform in translational cancer research and personalized cancer medicine. PDOs effectively retain the original tumor tissue’s cellular heterogeneity and genetic complexity, providing a physiologically relevant system for investigating tumor progression, therapeutic responses, and mechanisms of drug resistance (12). Studies have shown that breast cancer PDOs can be developed from all molecular subtypes, demonstrating high similarity to the original tumor tissue (13, 14). In a study, Sachs et al. (2018) established a living biobank of breast cancer organoids derived from primary and metastatic breast cancer tissues across various molecular subtypes. The organoids retained key histological and genomic features of the primary tumors, reflecting tumor subtype-specific characteristics and heterogeneity. The study confirmed the potential of PDOs for long-term expansion, drug screening, and translational research applications (15). Further highlighting the clinical relevance of PDOs, Mazzucchelli et al. (2024) demonstrated the utility of PDOs as patient-specific preclinical models for tracking tumor evolution in response to neoadjuvant chemotherapy. By comparing organoids derived before and after treatment, they reveal how therapy drives phenotypic and molecular changes, including the emergence of more aggressive, stem-like, and chemoresistant cell populations (16). A critical aspect contributing to the physiological fidelity of these models is the incorporation of extracellular matrix (ECM) components, which provide structural support and establish a biochemical and biophysical microenvironment that closely mimics *in vivo* conditions (17). ECM significantly influences breast tumor cell behavior through its physical properties, such as stiffness and elasticity, which can impact cellular morphology, differentiation, and proliferation (18). Incorporating ECM components in breast cancer organoid cultures enhances their physiological relevance, making them valuable models for studying tumor biology and evaluating drug efficacy in preclinical research.

Despite these advances, a critical gap remains in the integration of comprehensive immunological profiling within breast cancer PDOs. Current organoid studies often overlook the inflammatory and immunoregulatory landscape of the TME, which is instrumental in shaping treatment outcomes. Furthermore, limited efforts have been made to systematically compare PDOs derived from different breast cancer subtypes in terms of their cellular architecture, cytokine environment, and potential to recapitulate native tumor complexity. Thus, a model that integrates histopathological fidelity with immune microenvironment profiling is needed to better understand tumor heterogeneity and improve therapeutic stratification. To address these gaps, the study focuses on creating a patient-derived organoid-based 3D microphysiological disease model to identify diverse cell populations, migration patterns, and cellular expression, which may aid in developing personalized therapies.

- We report here the development and characterization of PDO cultures from breast cancer tissue of different patients (ER, PR, HER2+).
- The research evaluates the ability of PDOs to recapitulate the histopathological characteristics of the original breast tumor, including cellular architecture, tissue organization, and phenotypic heterogeneity.
- Additionally, the study assesses the levels of pro-inflammatory and immunoregulatory cytokines within the developed PDOs to better understand the tumor immune microenvironment and its potential role in cancer progression and therapeutic response.
- The study aims to present a platform for personalized cancer research, enabling the investigation of patient-specific tumor biology to better understand tumor-to-tumor heterogeneity and its implications for disease progression.

## 2 Materials and methods

### 2.1 Materials

Cell culture media and supplements, including DMEM (Catalog No. 11965-092), Charcoal Stripped Fetal Bovine Serum (FBS) (Catalog No.12676-029), and Antibiotic-Antimycotic (100X) (Catalog No. 15240096), were sourced from Gibco. Dyes and antibodies used included LIVE/DEAD Viability/Cytotoxicity Kit (Catalog No. L3224), CD20 (Catalog No. 14-0209-82), CD24 (Catalog No. PA5-114975), CD34 (Catalog No. MA1-22646), CD45 (Catalog No. 14-9457-82), CD73 (Catalog No. 41-0200), CD90 (Catalog No. MA6-16671), CD105 (Catalog No. MA5-17041), Fibronectin (Catalog No. 14-9869-82), laminin-alpha 3 (Catalog No. MA5-24246), E-cadherin (Catalog No. 13-1700), Goat anti-Mouse IgG2a Alexa Fluor™ 594 (Catalog No. A21135), Alexa Fluor™ 488 phalloidin (Catalog No. A12379), and Goat anti-rabbit IgG2a Alexa Fluor™ 568 (Catalog No A78955). Geltrex™ Reduced-Growth Factor Basement-Membrane Matrix (Catalog No. A1413302) from Gibco. SuperBlock™ Blocking Buffer (Catalog No. 37515), Image-iT™ Fixative (Catalog No. I28700), Hoechst 33342 (Catalog No. H21492) and PBS, pH 7.4 (Catalog No. 10010023).

### 2.2 Tumor collection

Tumor sections from breast cancer patients were obtained from Chittaranjan National Cancer Institute (CNCI), Kolkata, India, following ethical approval (CNCI-IEC-JC3-2024-103) and informed consent from every patient. Tumor samples were collected during Modified Radical Mastectomy (MRM) procedures. The samples were collected in a sterile container and fixed in 10% buffered formalin for clinical reporting. For organoid culture, approximately 25–30 g of tumor tissue was carefully excised by an experienced pathologist under aseptic conditions in DMEM medium supplemented with 20% FBS and 1% anti-anti, and stored at 4°C until it was processed within 1 h.

### 2.3 Patient-derived organoid culture

Breast cancer PDOs were established using a mechanical dissociation-based culture method involving pipetting and slicing. In our study, we utilized a mechanically dissociation-mediated culture method involving gentle pipetting and slicing to process tumor tissues into small fragments. Mechanical dissociation is an alternative to enzymatic digestion that includes physically breaking down the tissue into smaller fragments or single cells. This can be accomplished through the use of techniques such as pipetting, shearing, or mincing. This method reduces the amount of disruption that occurs to cell-cell interactions and components of the extracellular matrix, both of which are potential factors that are essential for the preservation of tissue-specific traits. Helps to Maintain the Tissue Architecture: In contrast to enzymatic approaches, mechanical methods are more delicate and preserve the native tissue structure as well as the interactions between cells, which can be critical for certain types of organoids. The tumor tissues were washed thoroughly multiple times with PBS to remove debris and blood contaminants. The washed tissue sliced into small fragments using sterile surgical scissors and then filtered through a 40 µm cell strainer. The fragmented tissue pieces (3–4 mm) were transferred onto a 6-well plate containing coverslips, and basement membrane matrix hydrogel was carefully poured over the tissue fragments to embed them within a 3D matrix. The culture was incubated at 37°C for 20 min to allow the gel to stabilize. After solidification, 1 mL of organoid culture media was gently added and changed every 2–3 days. The cultures were maintained in a humidified incubator at 37°C with 5% CO_2_ for a period of 30 days. After optimal growth and migration of the heterocellular population, the PDOs were fixed in 4% paraformaldehyde and evaluated for morphological and immunostaining similarity (19).

### 2.4 Live/dead analysis

The PDOs were grown for 30 days and then transferred to a 6-well plate. The PDO domes were fixed with Image-iT™ Fixative for 20 min, then washed with PBS, and samples were blocked with a SuperBlock™ Blocking Buffer for 2 h. After washing with PBS, the PDOs were incubated with calcein and ethidium homodimer at 37°C for 45 min. The staining solution was then removed, followed by two additional PBS washes. Subsequently, the PDOs were incubated with Hoechst solution (H21492) for 10 min. After thorough PBS washing, the PDOs were mounted on slides and examined using a confocal microscope (Leica DMi8, STELLARIS 5).

### 2.5 Hematoxylin and eosin

The BC tissues and PDOs were processed overnight by an automated tissue processor, and then, the samples were embedded in paraffin wax in a cassette. Sliced were made from the wax block by microtome (3–5 microns) and collected over the mayor’s albumin-coated slide with proper labeling. After that, staining was done by an automated staining machine (LEICA) for H&E staining, and slides were analyzed.

### 2.6 Immunohistochemistry

The BC tissues and PDOs were sliced by microtome (3–5 microns) and mounted in a Poly-L-Lysine (PL) coated slide by LEICA. The slides were fixed with 4% paraformaldehyde, blocked overnight, and then followed by automated IHC staining protocols for ER, PR, HER2/neu, CD20 (1:250), CD24 (1:100), CD34 (1:250), CD45 (1: 250), CD73 (1:100), CD90 (1:500), CD105 (1:500), E-cadherin (1:1000), Fibronectin (1:500) and Laminin (6:500). Differential counter stains were done by Hematoxylin. The dilutions of the antibodies were used according to the manufacturer’s instructions.

### 2.7 Immunofluorescence

After optimal growth and migration of the heterocellular population, the PDOs were stored at 4°C, and migrated cells on the coverslip was fixed with Image-iT™ Fixative for 20 min. After PBS washing, fixed cells were blocked in SuperBlock™ Blocking Buffer for 2 h. Following blocking, cells were incubated overnight at 4°C with CD20 (1:250), CD24 (1:100), CD34 (1:250), CD45 (1: 250), CD73 (1:100), CD90 (1:500), CD105 (1:500), E-cadherin (1:1000), Fibronectin (1:500) and Laminin (6:500). Then, cells were washed two times with PBS and incubated for 3 h with secondary antibody goat anti-mouse IgG2a Alexa Fluor 594 (0.25:500). The cells were then washed with PBS, stained with Alexa Fluor 488 phalloidin for 20 min, followed by Hoechst 33258 staining for 10 min. After an additional PBS wash, the coverslips were mounted and visualized using a confocal microscope (Leica DMi8, STELLARIS 5).

### 2.8 Biochemical analysis

The PDO culture media were collected on day 14 and stored at −20°C until six samples were obtained for analysis. The levels of catalase (CAT), superoxide dismutase (SOD), glutathione (GSH), nitric oxide (NO), and lipid peroxidation were estimated using BT LAB assay kits, following the manufacturer’s protocols. Briefly, the media were thawed, and colorimetric assays were performed in triplicate. Absorbance was recorded at 400 nm and 550 nm using a microplate reader, and data were analyzed using standard curves and control samples.

### 2.9 Cytokine profiling

The collected media from different PDO groups were analyzed to quantify cytokine levels, including interleukin-6 (IL-6), interleukin-8 (IL-8), Tumor necrosis factor alpha (TNF-α), Interferon‐gamma (IFN‐γ), and Transforming growth factor-β (TGF-β). The experiment aimed to assess cytokine secretion from PDOs after culturing them for 28 days. A solid-phase sandwich ELISA was employed, utilizing pre-coated plates with target-specific capture antibodies. According to the manufacturer’s protocol, samples, standards, streptavidin-HRP conjugate, chromogenic substrate, and stop solution were sequentially added. The final absorbance was recorded at 450 nm and 550 nm using a microplate reader.

### 2.10 GO and KEGG pathway enrichment analysis

GO (http://www.geneontology.org) and KEGG (Kyoto Encyclopedia of Genes and Genomes) database pathway enrichment analyses were carried out with the assistance of Metascape. These analyses concentrated on enriching the genes that were discovered by cytokine microarray analysis. KEGG is a tool that assists in the elucidation of various molecular machinery from the perspectives of genomic, proteomic, chemical, and systemic functional aspects (https://metascape.org/gp/index.html#/main/step1). The variable ontologies that are depicted in gene ontology databases are those that define the essential features of genes and gene products. In the case of Homo sapiens, a p-value of less than 0.05 was deemed to be statistically significant. The linked genes were visualized through the use of heatmap analysis, which was undertaken for the purpose of data analysis.

### 2.11 Protein−protein interaction network construction

For both the construction and the analysis of the networks, the Cytoscape program, version 3.8.0, was utilized. Connectivity for the building network in Cytoscape, which is provided by Genemania (http://genemania.org/), assists in the prediction of protein-protein interactions and the network pharmacology of various cytokines. In the end, we were able to determine the top targets that had node degrees that were relatively high. The prediction of PPI networks was accomplished by the utilization of searching tools for interacting genes (STRING; http://string-db.org) (version 10.0). There were a total of 30 edges and 22 connective nodes that made up the PPI network.

### 2.12 Statistical analysis

For the purpose of carrying out a one-way analysis of variance (ANOVA), GraphPad Prism 6, version 6.07 was utilized. The data was averaged for three repeats and error bars represent their standard deviation (mean ± SD; n = 3) and the statistical significance was calculated using one-way ANOVA, followed by Dunnett’s multiple comparison test, (***p < 0.001). The probability at this level was determined to be significant. All of the experiments were carried out in triplicate and were performed twice, unless it was specifically stated otherwise.

## 3 Results

### 3.1 Establishment of breast cancer PDO harboring

**Figure 1:**
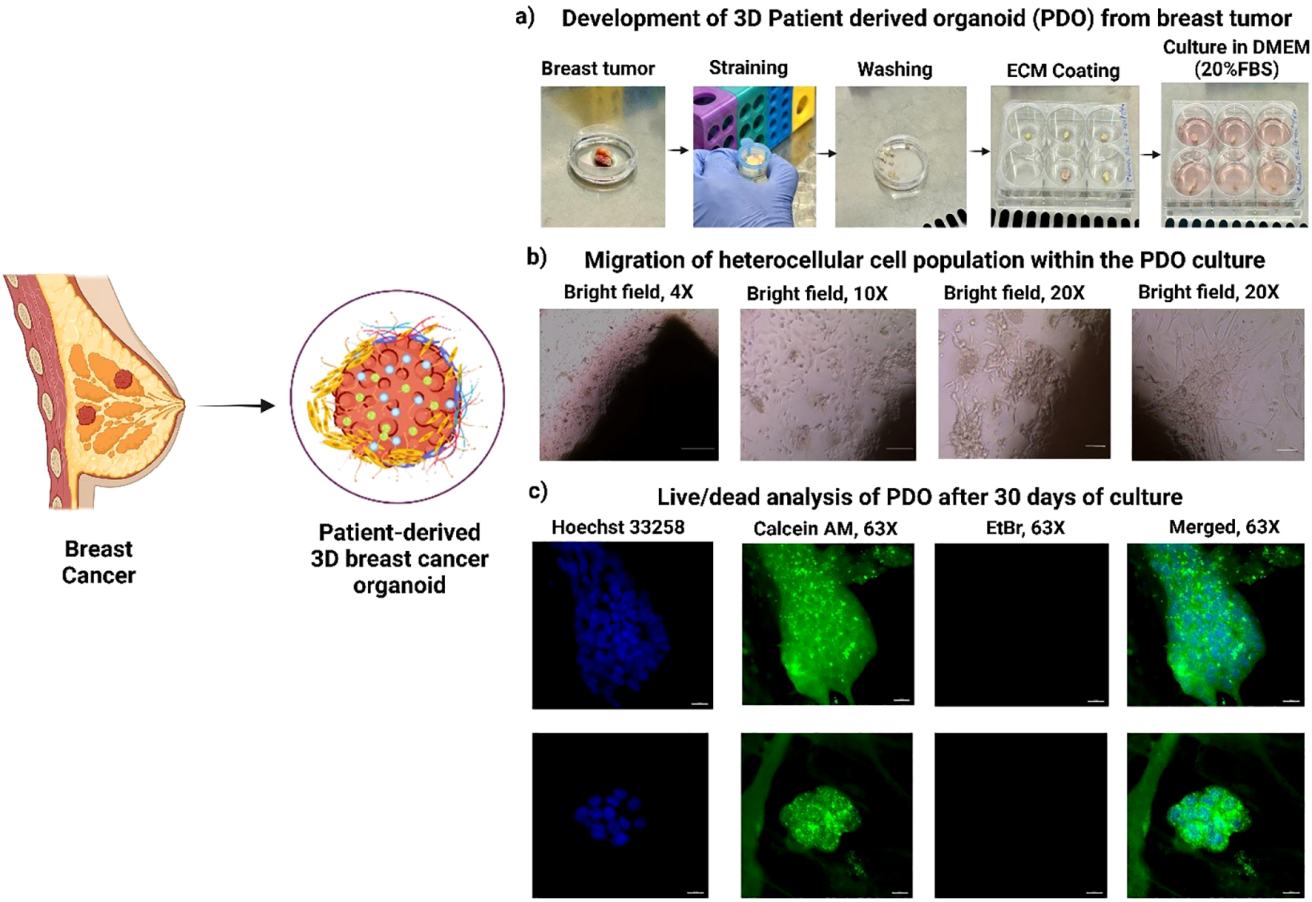
Development and characterization of 3D Patient-derived Organoids (PDOs) from Breast tumor tissues. **(a)** Schematic representation of PDO generation from patient breast tumor samples, **(b)** Migration of heterogeneous cell populations within PDOs, highlighting cellular dynamics, **(c)** Live/dead viability analysis of PDOs after 30 days of culture, demonstrating sustained cell viability.

The objective of this study was to establish a simple and effective method for PDO development from the biopsy specimens of breast cancer patients. The inclusion criteria for tumor collection were female patients aged 18 years and above with histologically confirmed breast cancer who were eligible for upfront surgery (prior to any chemotherapy), patients of all races and ethnicities; and ability to understand and willingness to provide written informed consent. The exclusion criteria included patients with any untreated active infection, and patients who had received any form of chemotherapy prior to surgery. The study was designed to evaluate the reliability of PDOs in accurately replicating breast cancer characteristics. The biopsy samples were received from the CNCI, and using the above-described method, PDOs were successfully grown from breast tumor tissues in DMEM medium supplemented with 20% FBS and 1% anti-anti at 37°C in a 5% CO_2_/95% air humidified incubator (Thermo HERA cell, USA). The culture was maintained for 30 days to ensure optimal growth and migration of the heterocellular cell population, **Figures 1a, b**. Migration was observed to initiate between days 4 to 7 across all PDO samples. Notably, a similar sequential migration pattern was observed, beginning with fibroblast cell migration, followed by endothelial cells and, subsequently, stem cells (20). Studies revealed that in breast cancer, CAFs and EMT-induced tumor cells migrate first due to their enhanced mobility and ECM remodeling abilities (21). We observed a similar migration pattern, where fibroblasts were the first to migrate, likely due to their role in ECM remodeling and secretion of pro-migratory factors, TGF-β and IL-6, **Supplementary Figure S1**. Next, immune cells including, T cells, and macrophages, alongside epithelial and endothelial cells, migrate through a combination of chemokine signaling, cytokine gradients, and ECM remodeling (22). Following fibroblast migration, cancer stem cells (CSCs) exhibit high motility and invasive potential, contributing to tumor heterogeneity. A study revealed that CSC responds to fibroblast-derived signals via activation of Wnt and Notch signaling pathways and stimulates migration (23).

### 3.2 Live/dead analysis of cultured PDO

It is possible to re-create the lobular structure of mammary gland organoids as a branched network of ducts that terminate in spherical alveoli or lobules. This structure is a component of the terminal ductal lobular unit (TDLU). These lobular structures contain ducts that branch off and terminate in these organoids, which are a representation of the anatomy of the mammary gland in wild-type mammals. The PDOs were successfully cultured for 30 days, and their cellular integrity and viability were evaluated using live/dead dual staining. The confocal microscopy images demonstrated a high percentage of viable cells, as indicated by strong green fluorescence from calcein staining due to higher esterase activity (increased calcein-AM binding), **Figure 1c**. Additionally, the absence of red fluorescence from ethidium homodimer staining confirmed no cell death within PDO in 30-day culture. The lobular structure of the mammary gland are visible with a spherical end, which denotes that we can keep the pathophysiological and organ native cellular architecture. The results confirm the effectiveness of the culture conditions in sustaining PDO viability and structural integrity, highlighting their suitability for long-term *in vitro* studies and therapeutic applications (24).

### 3.3 H&E staining to identify heterogeneous cells

**Figure 2:**
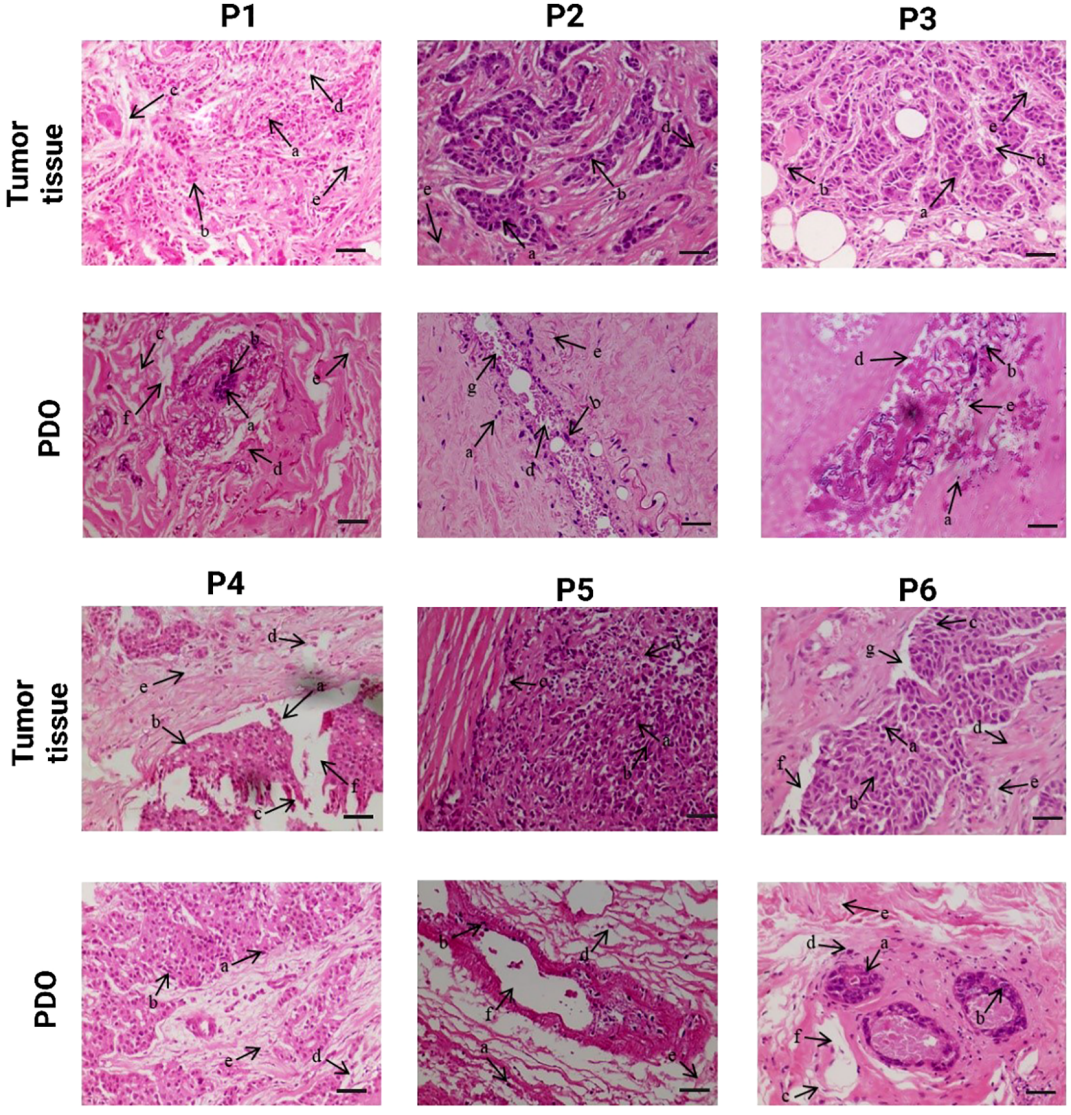
Comparative histological analysis of patient breast tumor and corresponding Patient-derived organoid (PDO), demonstrating the preservation of histological characteristics and cellular morphology in PDOs. The scale bar is 50 µm.

Histological characterization of breast tumor tissues and the developed PDOs were performed using H&E staining to evaluate the structural similarity to the original tumor and the preservation of cellular heterogeneity within the PDOs, **Figure 2**. Histological analysis demonstrated that PDOs closely recapitulated the structural and cellular features of their corresponding breast tumor tissues across all six patients (P1-P6). The analysis revealed a high degree of morphological similarity between tumors and their corresponding PDOs, confirming the organoids’ ability to preserve key histoarchitectural and cellular features. Both tumor tissues and PDOs consistently exhibited key components of the tumor microenvironment across patients P1-P6, including presence of luminal cells (a), myoepithelial cells (b), capillaries (c), fibroblast (d), and adipocytes (e). Luminal cells, typically associated with hormone receptor expression (ER and PR), reflect the intrinsic subtype of the tumor and were preserved in all PDOs, suggesting maintenance of hormone-responsive characteristics (25). Myoepithelial cells, which act as a barrier to invasion, were also retained, indicating preservation of the tissue’s basal structure; their loss is often linked to more aggressive tumor phenotypes (26). Fibroblasts, particularly CAFs, were present in both tumor and PDO sections and play a crucial role in ECM remodeling and promoting EMT. Their expansion is often driven by mesenchymal stem cells (MSCs) that upregulate markers such as VEGF, SDF-1, and TGF-β (27). Adipocytes, important stromal elements due to their proximity to mammary epithelium, were also observed and are known to influence tumor progression by secreting cytokines and adipokines that enhance cancer cell proliferation and invasion (28). Additionally, patient-specific features were preserved, indicating the ability of PDOs to capture inter-patient heterogeneity. Capillary structures (c), reflecting active angiogenesis, were seen in the tumors and PDOs of P1, P4, and P6, while interlobular (g) and large ducts (f) were present in the PDOs from P2, P4, P5, and P6, demonstrating the retention of complex ductal architecture (29). These results confirm that breast cancer PDOs reliably reproduce the histological characteristics of their original tumors, supporting their utility as robust *in vitro* models for disease modeling, therapeutic screening, and personalized medicine approaches.

### 3.4 Breast receptor expression (ER/PR/HER2) of cultured organoid to identify the specific subtype

**Figure 3:**
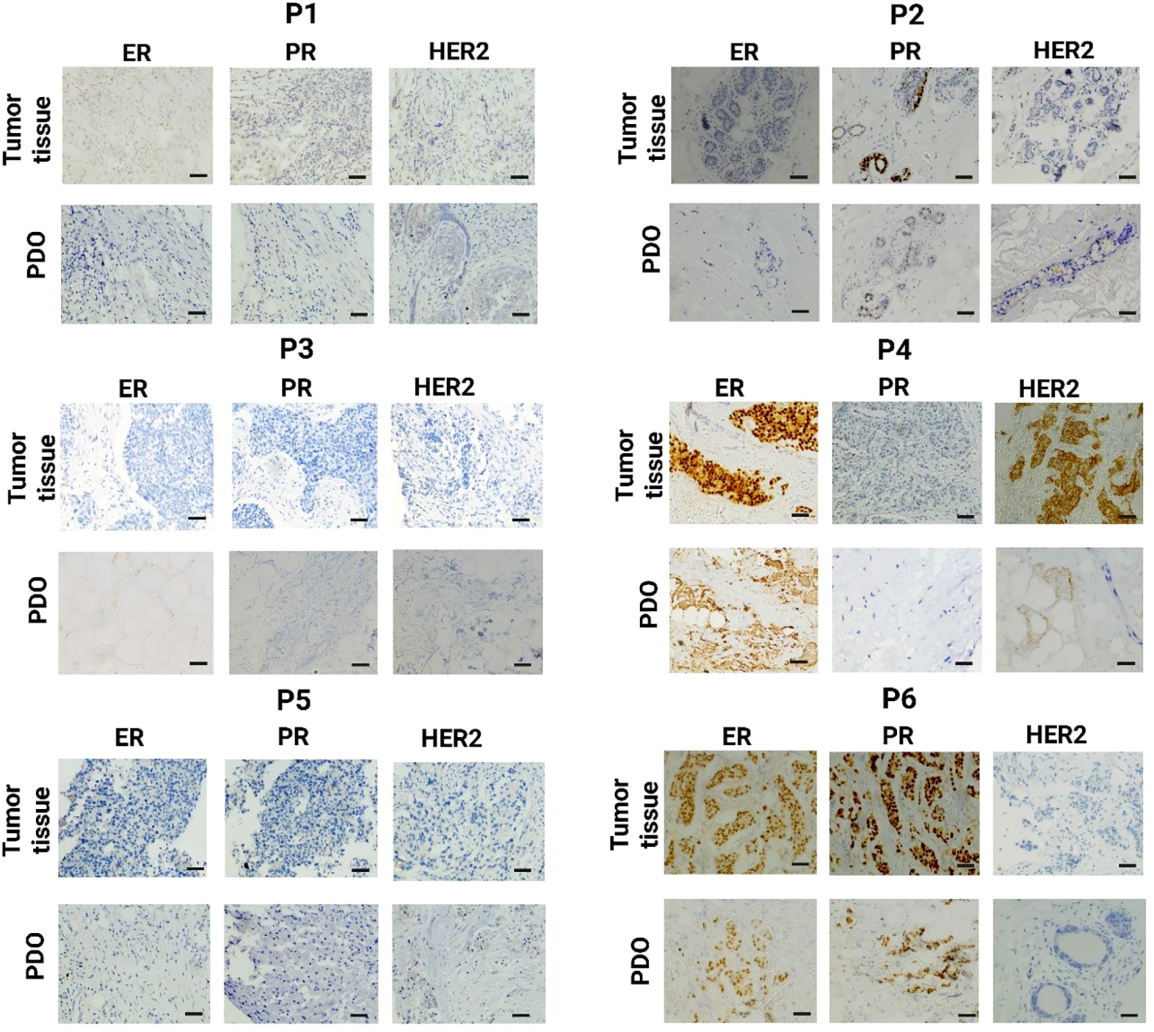
Immunohistochemistry (IHC) analysis of breast tumor tissue and the corresponding PDO derived from the same patient for estrogen receptor (ER), progesterone receptor (PR), and human epidermal growth factor receptor 2 (HER2) expression. The scale bar is 50 µm.

The IHC analysis of P1 revealed a total score of 0 for ER, PR, and HER2, indicating a complete absence of receptor expression. The PDO from P1 exhibited similar scores, with no detectable staining for ER, PR, or HER2, **Figure 3**. The ER and PR total 0 score indicates a nil percentage of positive cells with no staining intensity. Additionally, the 0 score for HER2 reflects no membrane staining within 10% of tumor cells. The P2 exhibited a 0 score for both ER and HER2. While the PR score was 1, indicating weak staining intensity. The PDO from P2 revealed the similarity. The P3 demonstrated a similar expression of breast cancer receptors like P1, with 0 scores for ER, PR, and HER2, indicating a complete absence of receptor expression. Similarly, the PDO from P3 exhibited the same scoring pattern, with no detectable staining for any of the three receptors. P4 revealed an ER total score of 8, with a proportion score of 5, indicating more than 67% positive cells, and an intensity score of 3, signifying high staining intensity. The PR score was 0, indicating no detectable expression. HER2 analysis revealed a score of 3+, characterized by high-intensity, complete membrane staining within more than 10% of tumor cells. Likewise, the PDO from P4 exhibited a similar receptor expression profile. The P5 demonstrated a similar expression of breast cancer receptors like P1 and P3, with 0 scores for ER, PR, and HER2. Similarly, the PDO from P5 exhibited the same scoring pattern, with no detectable staining for any of the three receptors. The findings suggest that the PDO accurately reflects the receptor-negative profile of the P5 tumor. For P6, the proportion score was 5, indicating more than 67% of positive cells, and the intensity score was 3, signifying high staining intensity for ER with a total score of 8. Though the intensity score was the same for PR, the proportion score was 2, indicating 1-10% positive cells. Whereas, HER2 was negative. Likewise, the PDO from P6 displayed an identical receptor expression pattern, confirming the structural and molecular similarities of PDOs in modeling tumor characteristics.

### 3.5 IHC analysis of cultured PDO

**Figure 4:**
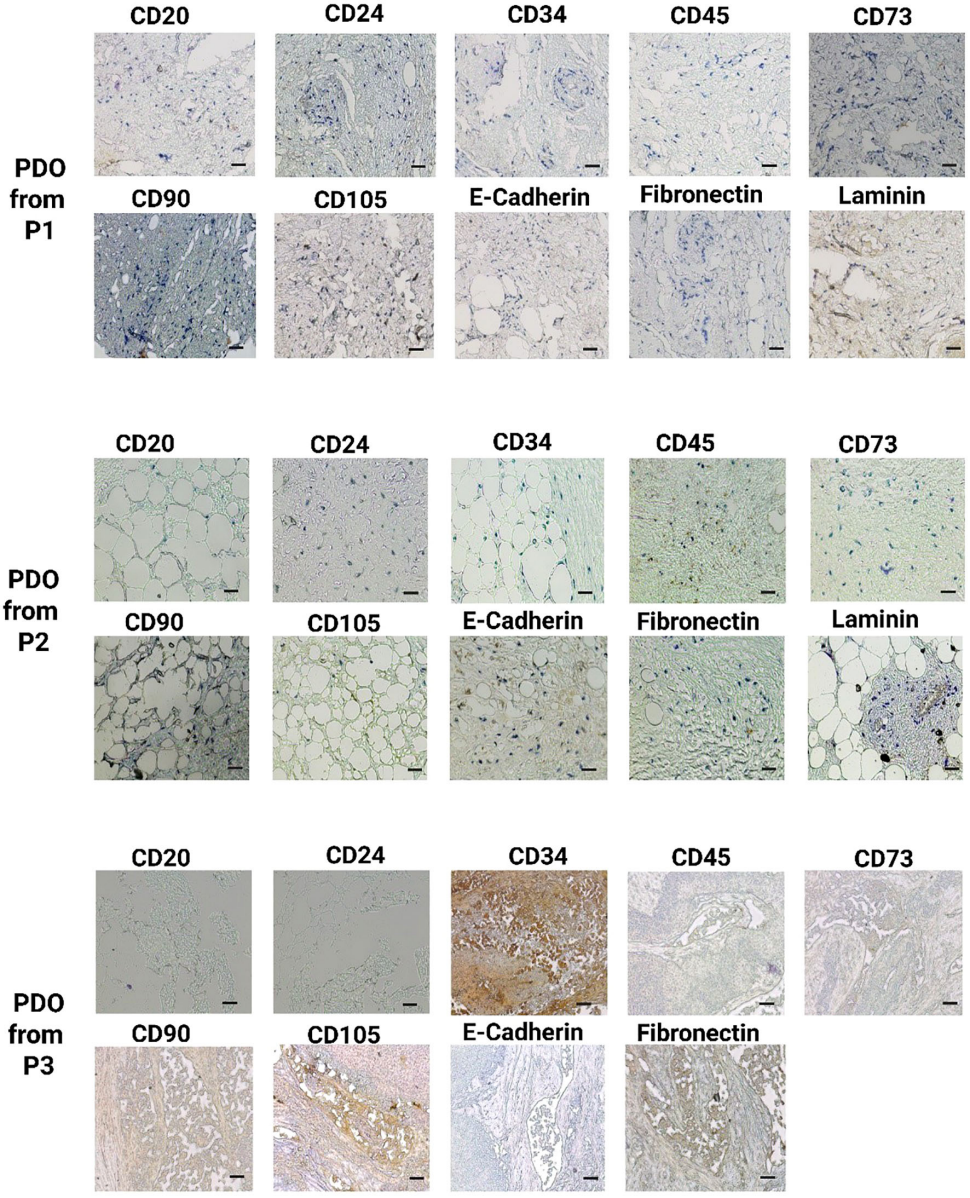
IHC analysis of key breast cancer biomarkers, including CD20 (B cells), CD24 (luminal epithelial cells), CD34 (vascular endothelial cells), CD45 (leukocyte infiltration), CD73, CD90, and CD105 (mesenchymal stem cells), E-cadherin (epithelial-to-mesenchymal transition), fibronectin, and laminin (fibroblast) in PDOs from patients P1, P2, and P3. The scale bar is 50 µm.

**Figure 5:**
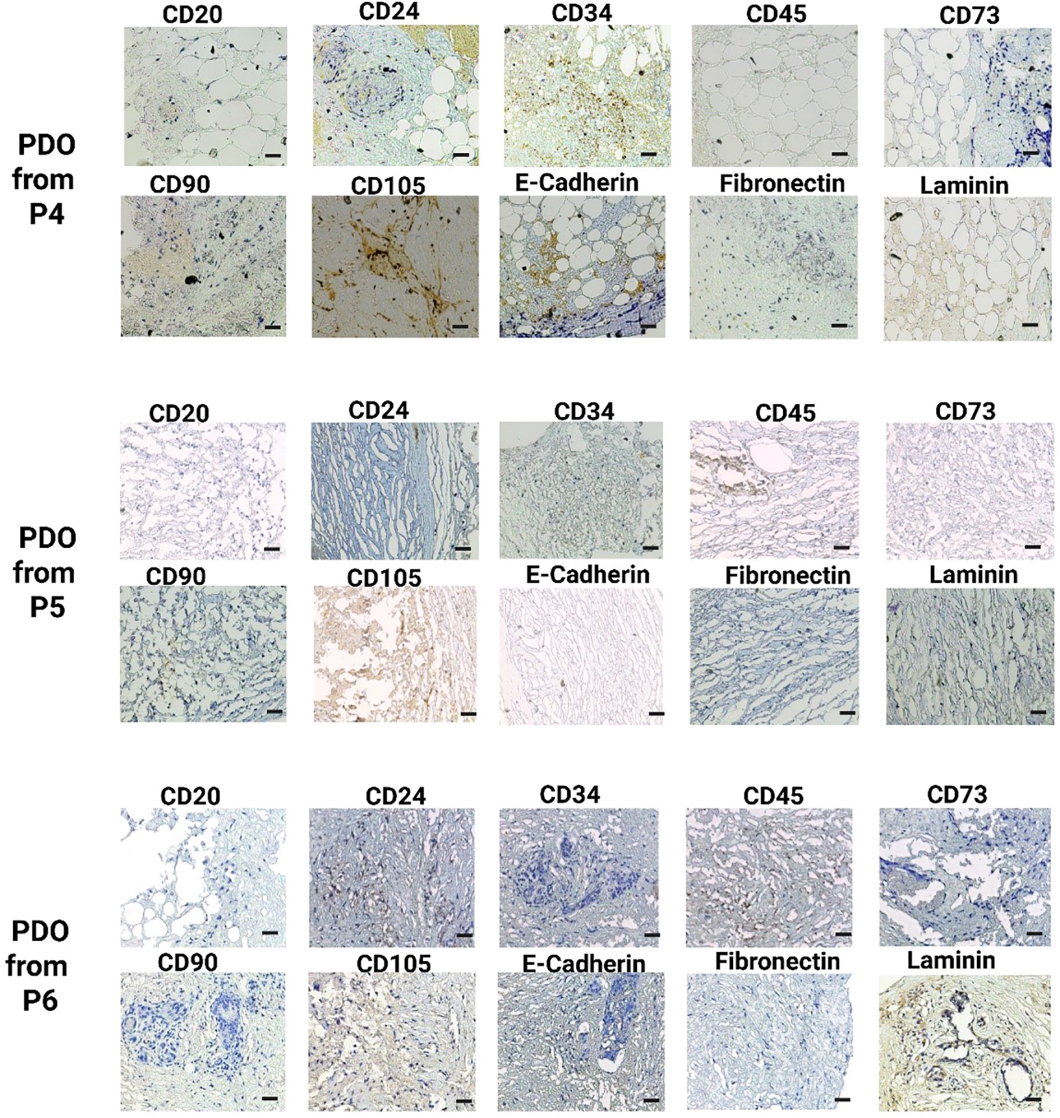
IHC analysis of key breast cancer biomarkers, including CD20 (B cells), CD24 (luminal epithelial cells), CD34 (vascular endothelial cells), CD45 (leukocyte infiltration), CD73, CD90, and CD105 (mesenchymal stem cells), E-cadherin (epithelial-to-mesenchymal transition), fibronectin, and laminin (fibroblast) in PDOs from patients P4, P5, and P6. The scale bar is 50 µm.

Subsequently, IHC staining was performed to compare the histological features of developed PDOs with their tumor tissues. We used a panel of ten different antibodies, including CD20, CD24, CD34, CD45, CD73, CD90, CD105, E-cadherin, fibronectin, and laminin, to identify different viable cell types in breast cancer PDOs, **Supplementary Table S1**. The IHC analysis demonstrated a significant elevation in the expression of these key markers, highlighting their crucial role in breast tumor progression and aggressiveness, **Figures 4** and **5**. CD20, a marker for B cells, has been correlated with the presence of tumor-infiltrating B cells, highlighting their role in immune modulation within the breast tumor microenvironment (30). A study revealed that the number of CD20+ B-cells was directly associated with HER2+ characteristics. Additionally, high CD20 was significantly linked with MMP-9 expression, a critical enzyme involved in ECM remodeling and tumor progression (31). The IHC analysis of PDOs from P1, P2, P4, P5, and P6 revealed high expression of CD20, whereas, in the PDOs from P2, the expression was patchy-positive and negative in P3, indicating heterogeneous B-cell infiltration. The results demonstrated that PDOs were able to retain the expression of CD20, similar to the original breast tumor.

CD24, a cell surface glycoprotein, was linked to tumor progression, metastasis, and cancer stem cell properties (32). The studies correlated the higher expression of CD24 with larger tumor size, axillary lymph node metastasis, and HER2+ status (33, 34). Additionally, the elevated CD24 expression was significantly associated with EMT by facilitating cellular plasticity and thus enhancing tumor invasiveness and metastasis (34). The IHC analysis of PDOs from P1, P2, P4, P5, and P6 revealed positive expression of CD24, whereas PDOs from P3 exhibited negative expression. The CD24+ expression in PDOs was linked to intra-tumor heterogeneity and high metastatic properties of breast cancer. CD34, a marker for hematopoietic stem cells (HSCs) and endothelial progenitor cells closely associated with angiogenesis (35). Elevated CD34 expression linked to increased endothelial cell proliferation, neovascularization, and enhanced tumor progression by directly promoting breast cancer cell proliferation, migration, and invasion (36). Studies correlated high expression of CD34 with the self-renewal and multi-differentiation potential of breast cancer stem cells (37). In this study, IHC analysis revealed high CD34 expression in PDOs from P1, P2, and P6, indicating a well-developed vascular network and active angiogenesis. In contrast, PDO from P5 and P6 exhibited patchy-positive CD34 expression, suggesting heterogeneous endothelial proliferation, while P3 showed no CD34 expression. The variation in CD34 expression across PDOs highlights the heterogeneity in tumor vascularization. CD45, a pan-leukocyte marker, identifies the immune cell infiltration within the breast tumor (38, 39). A study revealed that CD45+ cells, particularly tumor-associated macrophages (TAMs) and Regulatory T (TReg) cells, promote breast cancer aggressiveness through the JAK/STAT and NF-κB signaling pathways (40, 41). The elevated expression promotes chronic inflammation, EMT, and metastatic potential in breast cancer. The PDOs from P1, P2, P3, P4, P5, and P6 exhibited high CD45 expression, confirming the significant immune cell infiltration and an immunosuppressive tumor microenvironment with the PDOs.

CD73, CD90, and CD105 markers commonly associated with MSCs, were indicative of tumor plasticity and stemness in cancer (42). The breast tumor microenvironment was typically hypoxic, which facilitated tumor survival by promoting angiogenesis and metastasis. A study demonstrated that hypoxia-induced CD73 expression through the activation of hypoxia-inducible factor 1 (HIF-1) and CD73 serves as a trigger for EMT (43). Another study demonstrated that CD73 activates the PI3K/Akt signaling pathway, and thus enhances breast cancer cells proliferation, survival, and migration (34). Moreover, clinically elevated expression of CD73 was correlated with increased tumor aggressiveness and reduced patient survival (44). The IHC analysis of PDOs revealed CD73 positivity across all patient tumor samples, further validating its involvement in tumor growth and metastasis. Similarly, CD105+/CD90+ cells exhibited enhanced proliferative and migratory capacities. The higher migratory abilities were strongly associated with aberrant regulations of EMT (45, 46). Additionally, CD105 was linked to active neoangiogenesis, supporting breast tumor vascularization (47). The IHC analysis of PDOs from P1, P2, P4, P5, and P6 exhibited positive expression of CD90+/CD105+ subpopulation, suggesting their role in maintaining tumor heterogeneity, promoting angiogenesis, and enhancing metastatic potential. Whereas, the PDOs from P3 displayed a patchy expression pattern for both CD90 and CD105. These MSC markers contribute to a tumor-supportive microenvironment by modulating the PI3K/AKT signaling pathway, thereby promoting cell survival, proliferation, and invasion (48). Furthermore, loss of E-cadherin correlated with increased tumor invasiveness and metastasis in breast cancer (49). A study revealed that E-cadherin loss activates β-Catenin and induce EMT in breast cancer (50). Simultaneously, the upregulation of fibronectin and laminin was linked with ECM remodeling. Studies revealed that tumor-derived ECMs exhibit higher levels of collagen type I, fibronectin and laminin, and these markers provide a tumor-supportive microenvironment that contributes to tumor biological features (51). Notably, both primary and metastatic tumors in TNBC and HER2+ breast cancers exhibited elevated fibronectin expression (52). IHC analysis of PDOs from all patient samples demonstrated elevated expression of E-cadherin, fibronectin, and laminin, driving ECM structural modifications, thereby enhancing tumor cell migration and supporting metastatic potential. The findings suggest that PDOs successfully recapitulate key features of tumor aggressiveness, including cellular heterogeneity and vascularization.

### 3.6 Expression of specific protein in heterogeneous migrated cells from PDO

**Figure 6:**
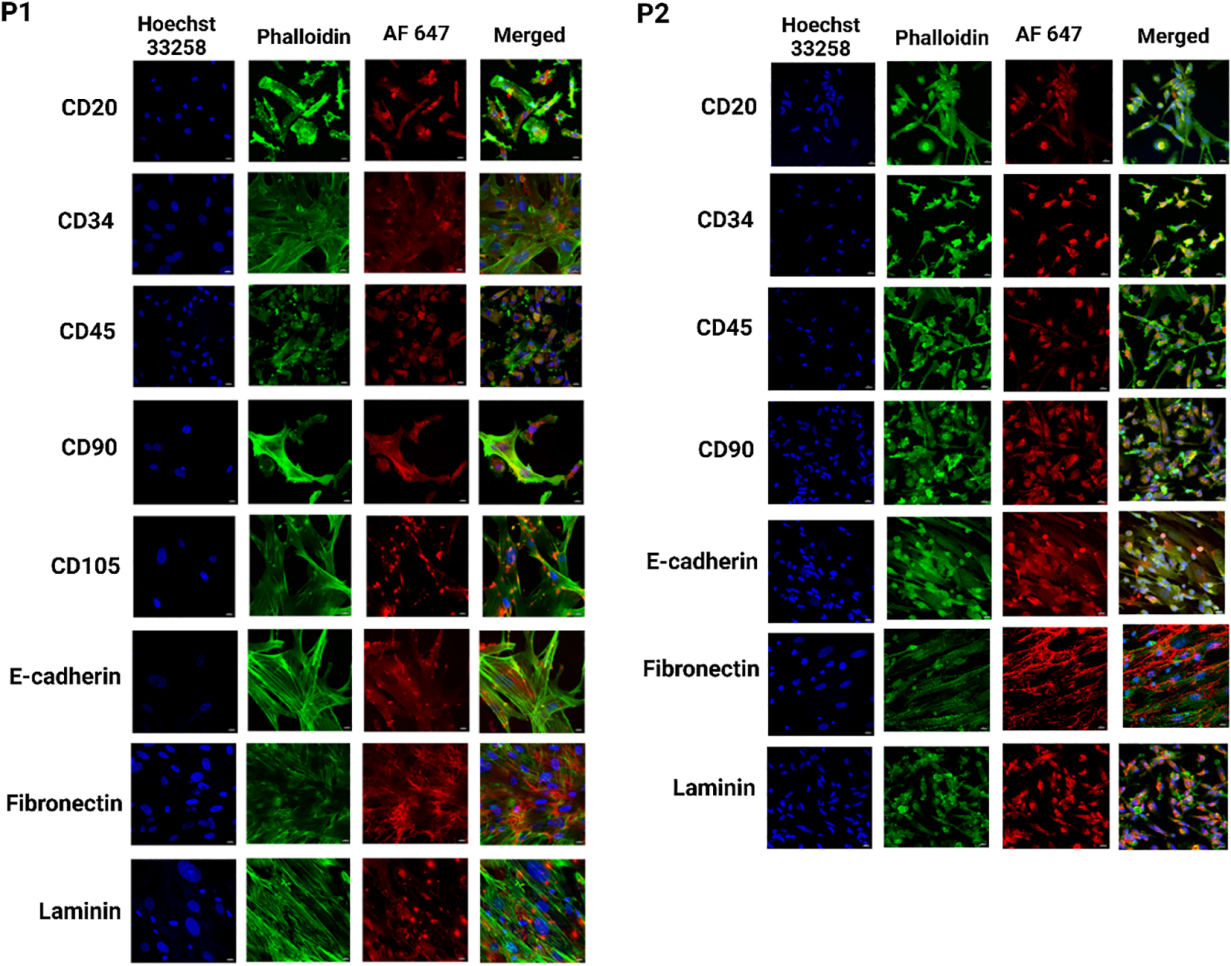
Identification of heterogeneous population within breast cancer PDOs via immunofluorescence to evaluate the expression of breast cancer-specific markers. PDO from P1 showed expression of CD20, CD34, CD45, CD90, CD105, E-cadherin, fibronectin, and laminin. PDO from P2 showed expression of CD20, CD34, CD45, CD90, E-cadherin, fibronectin, and laminin. The scale bar is 10 µm.

**Figure 7:**
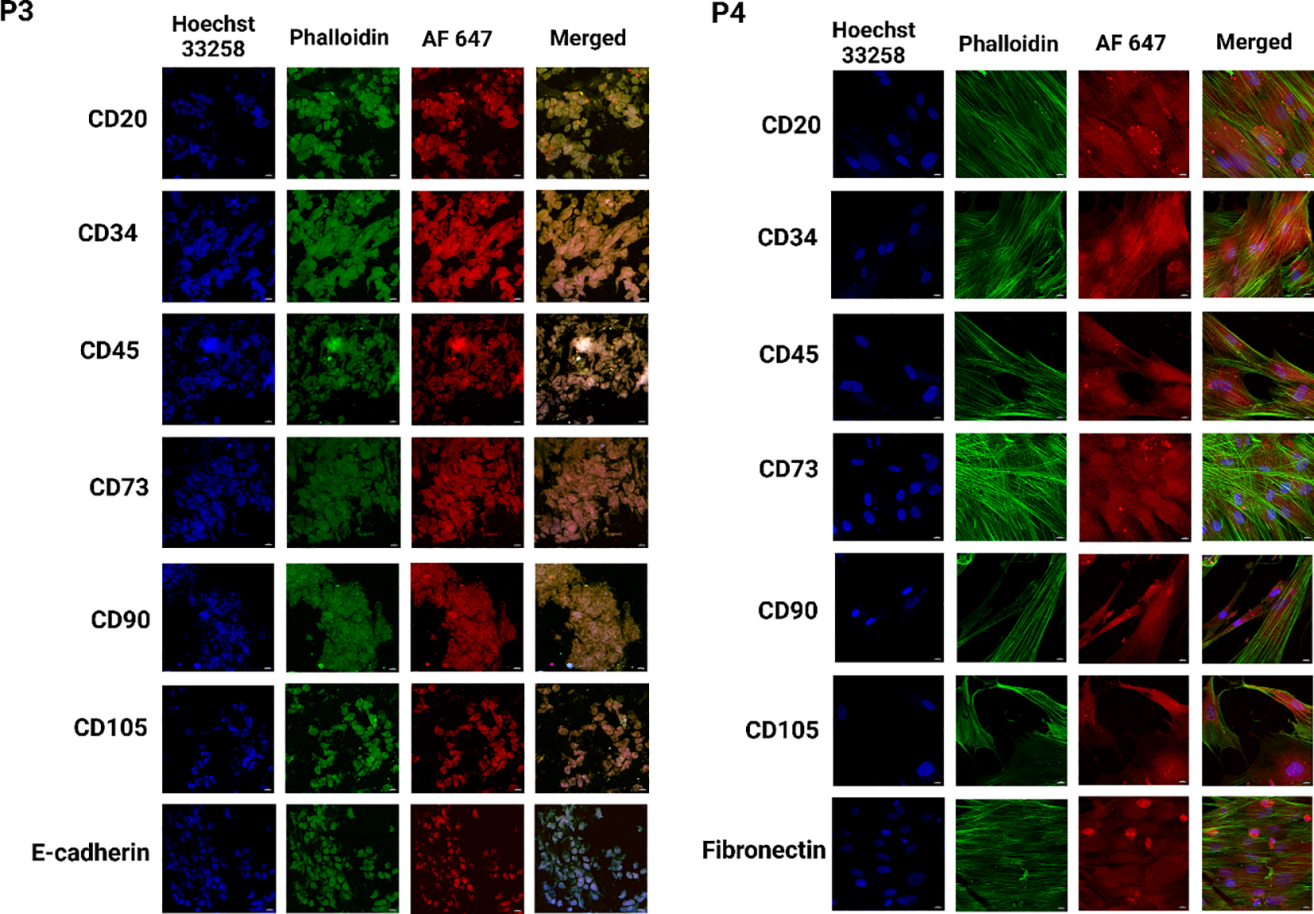
Identification of heterogeneous population within breast cancer PDOs via immunofluorescence to evaluate the expression of breast cancer-specific markers. PDO from P3 showed expression of CD20, CD34, CD45, CD73, CD90, CD105, and E-cadherin. PDO from P4 showed expression of CD20, CD34, CD45, CD73, CD90, CD105, and fibronectin. The scale bar is 10 µm.

We observed the dynamic migration of heterocellular populations within the PDO cultures. The distinct tumor cell subpopulations were identified by utilizing the panel of the above-mentioned antibodies specific to breast cancer biomarkers through IF, **Figures 6**, **7**). The confocal microscopic images showed CD20+ subpopulation in the PDOs from P1, P2, P3, and P4, confirming the presence of tumor-infiltrating B cells. CD20+ subpopulation, known to secrete cytokines and chemokines that activate the STAT3/NF-κB signaling pathway, leading to increased expression of pro-inflammatory mediators, including IL-6, TNF-and IFN-γ, and MMP-9, which facilitate tumor invasion and metastasis (53). Studies showed that the CD20+ population was more prevalent in the triple-negative and HER2+ breast cancer subtypes (54, 55). Furthermore, we observed positive expression for CD34 and CD45 in the PDOs from all patient samples. The CD34+ cells confirm the expression of fibroblasts, along with vascular and lymphatic endothelial cells, and CD45+ cells confirm the expression of leukocytes infiltrating cells in breast cancer (56). The findings demonstrated that PDOs were able to maintain the expression of CD34, and CD45 subpopulations to recapitulate the cellular heterogeneity of the breast tumors. Our analysis revealed that MSC-specific markers CD73, CD90, and CD105 were differentially expressed across all PDOs from various breast tumor samples. Notably, distinct CD73+ and CD90+/CD105+ subpopulations indicate the presence of MSC-like characteristics within the tumor microenvironment. MSCs have a remarkable capacity to differentiate into CAFs in response to a variety of soluble factors secreted by cancer cells. Notably, research has shown that transforming growth factor β1 (TGF-β1), derived from the tumor microenvironment, facilitates MSC to CAF differentiation (57). Interestingly, in this study, PDOs from P4 displayed a distinct cellular pattern, characterized by positive MSC marker expression along with an excessive fibroblast population. The observation suggests a more pronounced mesenchymal phenotype in P4, potentially driven by tumor-derived factors that promote fibroblast expansion and stromal remodeling. However, the differentiation of MSC-derived fibroblasts in breast cancer remains a controversial topic in cancer research. While studies have demonstrated the ability of MSCs to transition into CAFs, the extent to which these cells contribute to tumor progression and the mechanisms governing their differentiation remain unclear (58). A study identified a positive correlation between CD73 and EGFR expression, with CD73 promoting EGFR upregulation through the modulation of the transcription factor PPARγ (59). Furthermore, CD73 overexpression enhances the proliferation and migration via activating VEGF/Akt and EGFR/Akt signaling pathways (60). Here, we observed the CD73+ population in the PDOs from P3, suggesting massive lymph node involvement. Likewise, CD90+/CD105+ subpopulations increased EMT in breast cancer cells by cross-interacting with TGF-β/Smad and PI3K/Akt signaling pathways (61). The analysis revealed the expression of CD90+/CD105+ subpopulations in the PDOs from P1, P3, and P5, whereas P2 showed only the CD90+ population. Collectively, these markers CD73+ and CD90+/CD105+ promote tumor growth, angiogenesis, and metastasis in breast cancer cells, and positive expression of these markers in PDOs was an indicator of the MSC phenotype of the original tumor. E-cadherin, an EMT hallmark, promotes metastasis, and its loss increases breast cancer cell invasion. Additionally, associated with the upregulation of genes involved in TGF-β, ROS, and apoptosis signaling pathways, highlighting a shift toward a more aggressive tumor phenotype (62). Studies showed that E-cadherin contributes to a hyper-proliferative phenotype in breast cancer through its interaction with the transmembrane receptor EGFR (63). Here, we observed downregulated expression for E-cadherin in the PDOs from P4. In contrast, the expression was comparatively high in the PDOs from P1, P2, and P3, suggesting potential variability in the cellular characteristics and aggressive nature of the tumors among different patient samples. Laminin within ECM supports cell attachment and viability, aiding in the self-organization of primary breast cancer cells into tumoroids (64). A study discovered that tumor-derived ECM displayed higher levels of procollagen I, fibronectin, and laminin compared to normal breast tissue-derived ECMs. In TNBC and HER2+ breast cancer, high-level expression of fibronectin was strongly associated with reduced patient survival (52). Fibronectin also induces MMP2 expression, which contributes to ECM degradation. Additionally, it promotes cell invasion and metastasis by inducing EMT and activating crucial signaling pathways, including FAK, ILK, ERK, PI3K, and NF-κB (65). The confocal microscopy revealed the upregulation of laminin expression in PDOs from P1 and P2, while fibronectin expression was elevated in PDOs from P1, P2, and P4, confirming the ECM remodeling within PDOs to provide a tumor-supportive microenvironment for the growth of different tumor cells.

### 3.7 Exploration of oxidative defense mechanism

**Figure 8:**
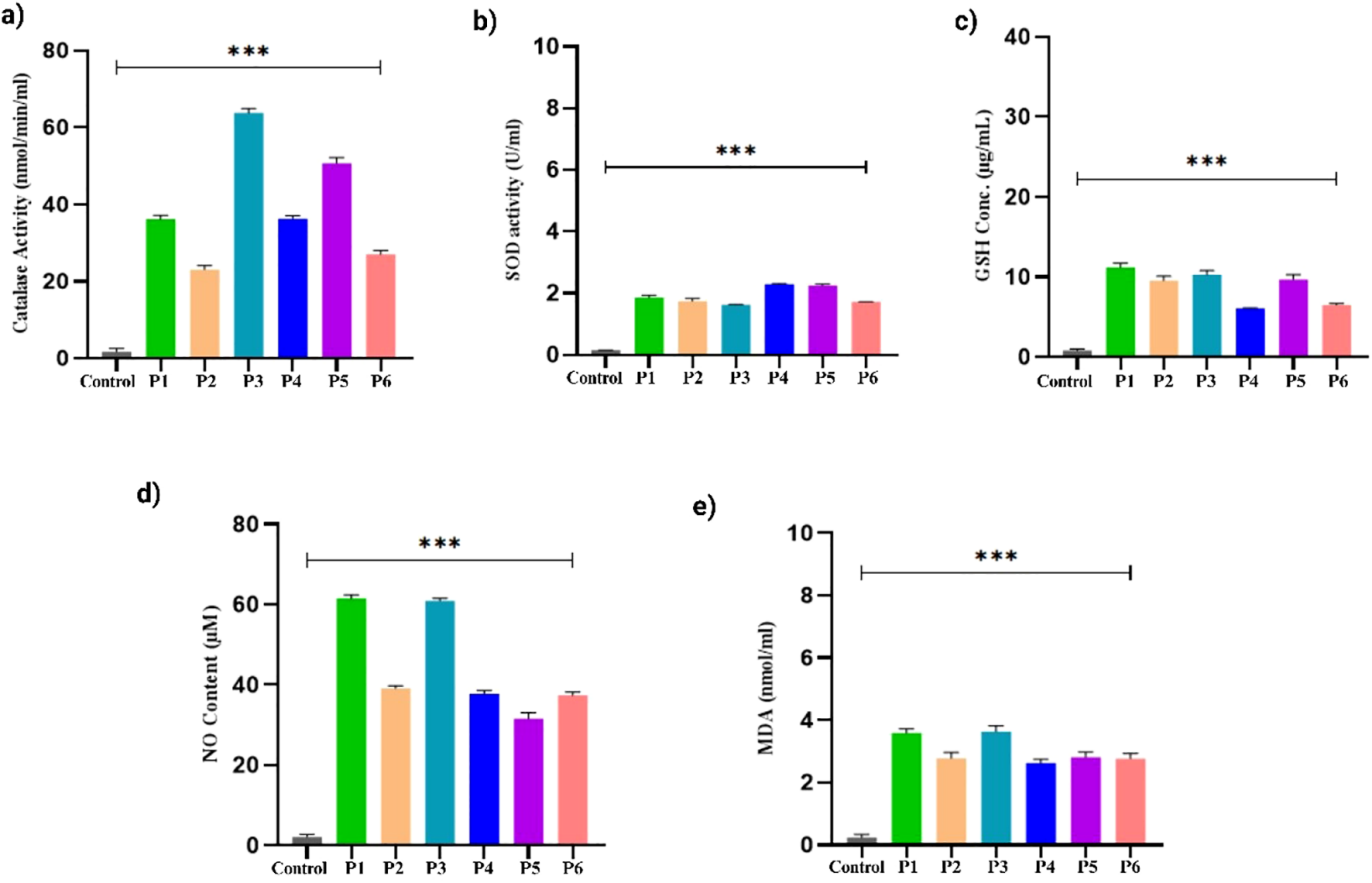
Oxidative stress markers levels in the breast cancer PDOs from different patients. **(a)** Catalase, **(b)** SOD, **(c)** GSH, **(d)** Nitric oxide, and **(e)** Lipid peroxidation. The data was averaged for three repeats and error bars represent their standard deviation (mean ± SD; n = 3) and the statistical significance was calculated using one-way ANOVA, followed by Dunnett’s multiple comparison test, (***p < 0.001).

Next, we evaluated the levels of the antioxidant defense system, including CAT, SOD, GSH, NO, and lipid peroxidation, in PDOs derived from different breast cancer samples. We observed elevated levels of CAT across all PDO cultures. Studies showed lower CAT activity was associated with increased breast tumor metastasis (66). While certain studies reveal that tumor cells can overexpress CAT to mitigate excessive hydrogen peroxide generated during rapid metabolic activity, thereby enhancing cancer cell survival (67). We observed low CAT levels in PDOs from P1, P2, P4, and P6, while PDOs from P3 and P5 exhibited comparatively higher CAT levels, **Figure 8a**. Additionally, we observed reduced SOD levels in the PDOs from different patient samples **Figure 8b**. Specifically, P1, P2, P3, and P6 show lower values, whereas P4 and P5 exhibit comparatively higher levels. Studies revealed that tumor progression and SOD activity were impaired due to inactivation by c-Jun and p53 signaling pathways, leading to excessive ROS accumulation. This oxidative stress induces DNA damage and promotes mutagenesis, thereby enhancing tumorigenic potential (68). Next, we recorded lower GSH levels in the PDOs, suggesting increased oxidative stress. The most significant decline in GSH levels was observed in P1 and P3 compared to P2, P4, P5, and P6, **Figure 8c**. Studies have established an inverse relationship between GSH and NO levels in breast cancer, where reduced GSH contributes to increased oxidative stress and NO accumulation (69). Notably, we observed elevated NO levels across all PDOs, with the highest increase in P1 and P3, while P2, P4, P5, and P6 exhibited comparatively lower but upregulated, **Figure 8d**. Furthermore, lipid peroxidation levels were significantly elevated in the PDOs, confirming oxidative damage. The PDOs from P2, P4, and P5 exhibited lower lipid peroxidation levels compared to P1, P3, and P6, suggesting a progressive increase in oxidative stress **Figure 8e**. Studies demonstrated that higher levels of lipid peroxidation profile in breast cancer patients promote tumorigenesis and metastasis (70, 71).

### 3.8 Secretome profiling

**Figure 9:**
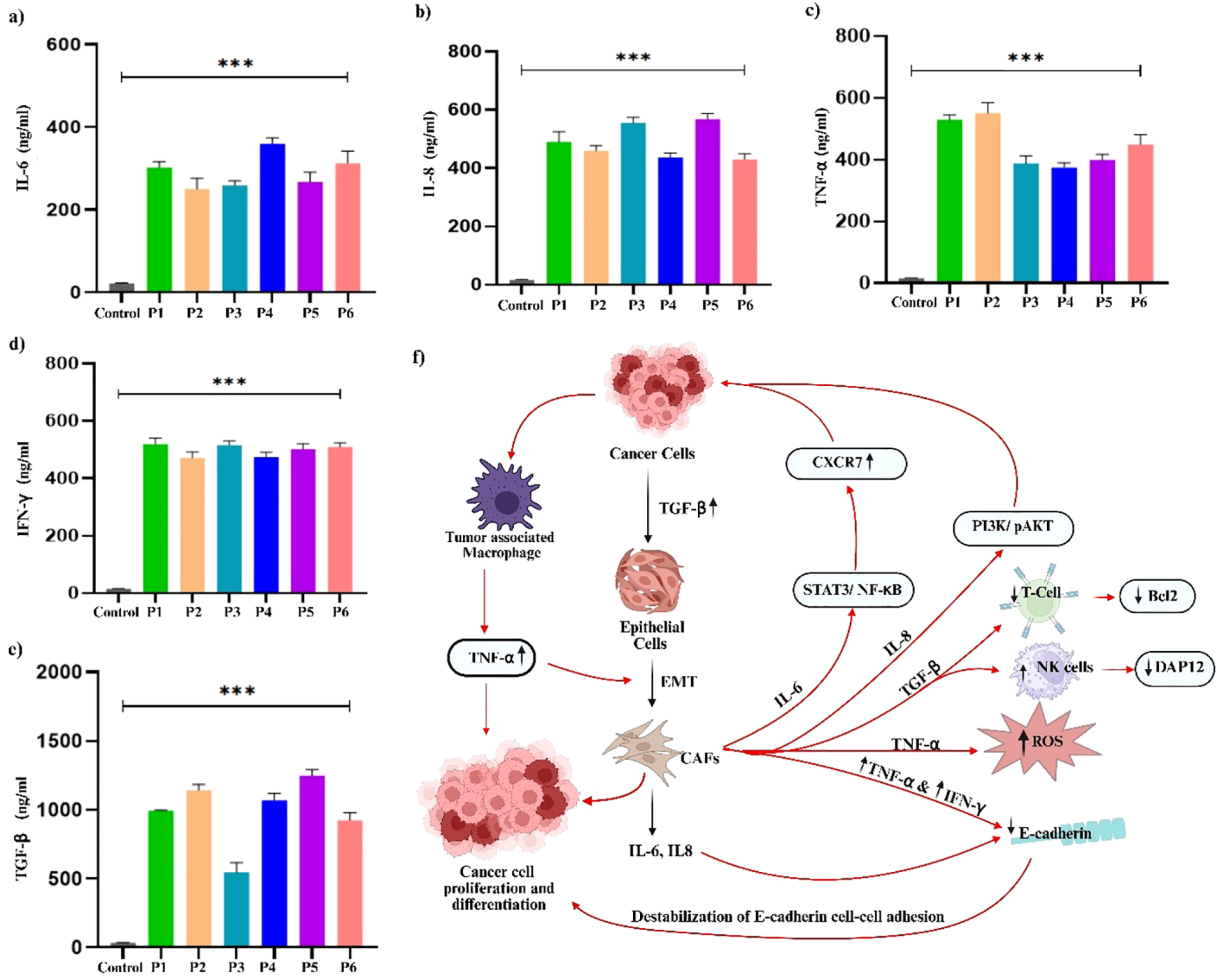
Analysis of pro-inflammatory and immunoregulatory cytokine prolife in breast cancer PDOs from different patients. **(a)** IL-6, **(b)** IL-8, **(c)** TNF-α, **(d)** IFN-γ, and **(e)** TGF-β. The schematic representation of the interconnected roles of **(f)** IL-6, IL-8, TNF-α, IFN-γ, and TGF-β in promoting cancer cell proliferation, differentiation, and tumor progression within the tumor microenvironment. The data was averaged for three repeats and error bars represent their standard deviation (mean ± SD; n = 3) and the statistical significance was calculated using one-way ANOVA, followed by Dunnett’s multiple comparison test, (***p < 0.001).

**Figure 10:**
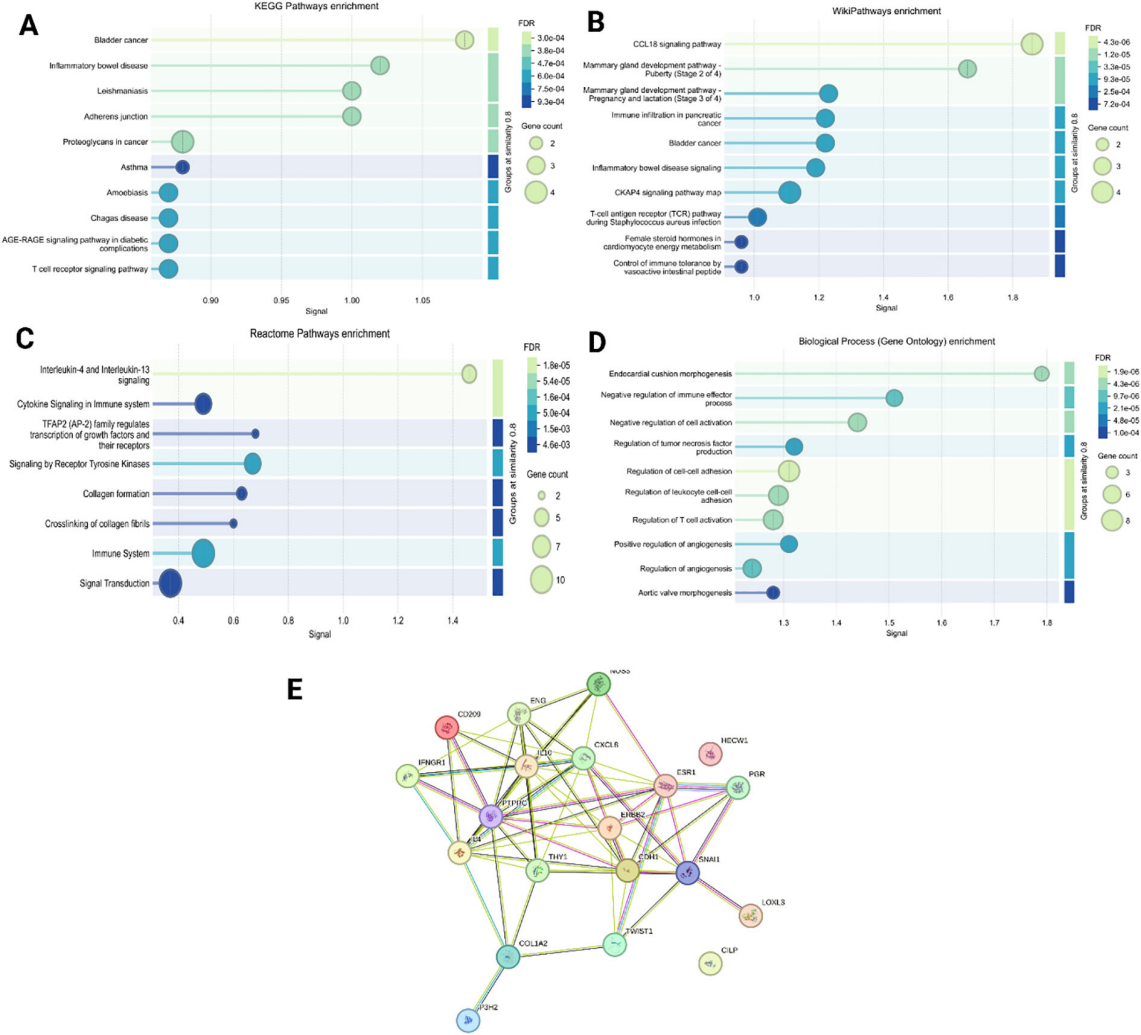
To identify various differently expressed proteins in the 3D organoid culture, we constructed PPI network by uploading the up- and downregulated proteins into **(A)** KEGG pathway enrichment, **(B)** Wiki pathway enrichment, **(C)** Reactome pathway, **(D)** Biological process (gene ontology) enrichment, and **(E)** STRING analysis.

Cytokine profiling of PDOs revealed significant alterations in key pro-inflammatory and immunoregulatory cytokines, including IL-6, IL-8, TNF-α, IFN-γ, and TGF- β, **Figure 9**. The levels of IL-6 were markedly upregulated in all PDO samples, with the highest increase in P1, P4, and P6, compared to P2, P3, and P5. The upregulated IL-6 expression promotes EMT through the JAK/STAT3 signaling pathway, thereby suppresses E-Cadherin expression, and facilitates breast tumor cell migration and invasion (72). Furthermore, the significant upregulation of IL-8 was associated with increased cancer cell invasiveness and a shift towards a mesenchymal phenotype. The IL-8/IL-8R axis has been shown to be essential for the maintenance of the invasive and mesenchymal phenotypes of breast cancer (73). We observed increased IL-8 expression in PDOs, with the highest levels detected in P1, P3, and P5, whereas P2, P4, and P6 exhibited relatively lower expression, confirming the aggressive and invasive characteristics of PDOs. Likewise, upregulated TNF-α contributes to EMT induction and invasion by stabilizing β-catenin and Snail through the NF-kB and PI3/AKT signaling pathways (74). Recently, a study showed that TNF-α and IFN-γ enhance the cancer cells invasiveness by downregulating the expression of E-cadherin (75). Our analysis revealed elevated levels of both TNF-α and IFN-γ, particularly in P1, P2, and P6, correlating with increased EMT and invasive potential of PDOs. The levels of TGF-β were found to be elevated in the PDOs, with the highest expression observed in P1, P2, P4, P5, and P6 when compared to P3. TGF-β is well-documented to play a dual role in cancer, functioning as a tumor suppressor during the early stages by inducing cell cycle arrest and apoptosis, while promoting tumor progression in later stages by facilitating EMT, invasion, metastasis, and immune evasion. The observed upregulation of TGF-β in these PDOs, which were derived from advanced breast cancer tissues, likely reflects its pro-tumorigenic function. Mechanistically, TGF-β has been shown to induce EMT by upregulating the transcription factor ZEB1, which in turn represses epithelial splicing regulatory proteins (ESRP1/2), key regulators of the epithelial phenotype. The downregulation of ESRPs contributes to the shift toward a mesenchymal, invasive phenotype in breast cancer cells. Additionally, TGF-β exerted immunosuppressive effects by downregulating T-cell activity, decreasing Bcl-2 expression, and impairing NK cell function, facilitating immune evasion (76). Collectively, the PDOs were observed with elevated pro-inflammatory and immunoregulatory cytokine profiles, confirming their resemblance to the breast tumor microenvironment. To explore this further, we constructed PPI networks by uploading the upregulated and downregulated proteins into STRING (C) and further analyzed by (A) GO, (B) KEGG (**Figure 10**).

## 4 Conclusion

In this study, we successfully developed and thoroughly characterized patient-derived organoids (PDOs) from surgically resected breast cancer tissues across a wide range of molecular subtypes. Our results indicate that these PDOs accurately replicate the histological architecture, phenotypic variability, and molecular profiles inherent to their corresponding primary tumors, as evidenced by the retention of key lineage-specific markers and a heterogeneous cellular composition. Notably, the PDO secretome displayed elevated oxidative stress markers and a distinct cytokine profile, which closely mirroring the complexity of the native tumor microenvironment. This emphasizes the physiological relevance of our PDOs and their enhanced capability to model tumor heterogeneity compared to conventional *in vitro* systems. A significant innovation of our platform is its capacity to integrate extensive molecular and immunological profiling with subtype-specific PDO generation. Unlike many previously established PDO protocols, which primarily utilize Matrigel and focus on morphological fidelity or a single subtype, our method allows for the efficient isolation and maintenance of PDOs from multiple clinically relevant breast cancer subtypes, such as luminal, HER2-enriched, and triple-negative breast cancers in basement membrane matrix hydrogel. This broad applicability is achieved through optimized protocols from tissue collection, ECM selection, and culture conditions, leading to high establishment rates and robust reproducibility across diverse patient samples. Our analyses reveal that these PDOs not only recapitulate the genomic, transcriptomic, and metabolic heterogeneity of original tumors, but also display elevated oxidative stress biomarkers and a distinct cytokine profile in the secretome, mirroring the complex dynamics of the breast tumor TME. Elevated oxidative stress markers in breast cancer PDOs directly reflect and drive TME remodeling by activating CAFs, which then secrete elevated levels of growth factors, cytokines, and matrix metalloproteinases, further fueling tumor growth, invasion, and angiogenesis. Additionally, our methodology incorporates immunological profiling, allowing detailed assessment of immune cell infiltration and cytokine dynamics within the PDO microenvironment. This approach not only enhances the functional relevance of the PDOs but also provides a comprehensive, patient-relevant model for investigating tumor-immune interactions and assessing therapeutic responses. Our methodology demonstrated high efficiency and reproducibility, with successful PDOs development achieved in over 70% of processed patient samples. The streamlined process from tissue collection to organoid development enables consistent and scalable PDO production within clinically relevant timeframes. This scalability positions our platform favorably for high-throughput drug screening and biomarker discovery, facilitating its integration into precision oncology frameworks and clinical workflows. In summary, this study presents a robust, subtype-specific, and immunologically informed platform for the development and characterization of breast cancer PDOs. Through the incorporation of molecular subtyping and immune profiling, this platform provides a more comprehensive and functionally relevant *in vitro* model for translational breast cancer research. The demonstrated efficiency, reproducibility, and compatibility with high-throughput and clinical applications underscore the potential of our platform as a predictive tool for the development of personalized therapies. Collectively, these advancements establish a strong foundation for the integration of PDOs into translational research and precision oncology, ultimately paving the way for individualized treatment strategies and enhanced clinical outcomes for breast cancer patients.

## Data Availability

The original contributions presented in the study are included in the article/**Supplementary Material**. Further inquiries can be directed to the corresponding authors.
