# Supplementary material for: Mechanically induced development and maturation of 3D in-vitro organoid platform: an organotypic heterogeneous microphysiological model of patient-derived organoids with ER/PR/HER2+ breast cancer
Source: Front Immunol. 2025 Jul 31;16:1594405. doi: 10.3389/fimmu.2025.1594405 (PMC12350514; doi:10.3389/fimmu.2025.1594405)
Supplement: Supplementary file 1 [file DataSheet1.pdf]

Supplementary Information

Mechanically induced development and maturation of 3D in-vitro organoid platform: An organotypic heterogeneous microphysiological model of patient-derived organoids with ER/PR/HER2+ breast cancer.

Mamta Kumari[1], Kamare Alam[2], Anamitra Bhattacharya[2], Nakka Sharmila Roy[2], Vaishnavi Madhasu[2], Bitan Guchhait[2], Sangita Dan[3], Soma Sett[3], Jayanta Chakrabarti[3], Chandan Mondal[4*], Velayutham Ravichandiran[5*], Subhadeep Roy[2*]

[1]Department of Pharmaceutics, National Institute of Pharmaceutical Education and Research, Kolkata, West Bengal, India, 700054.
[2]Department of Pharmacology and Toxicology, National Institute of Pharmaceutical Education and Research, Kolkata, West Bengal 700054, India.
[3]Surgical Oncology, Chittaranjan National Cancer Institute, Kolkata., 700160, India
[4]Molecular pathology, Department of Laboratory of Medicine, Chittaranjan National Cancer Institute, Kolkata. 700160, India
[5]Department of Natural Products, National Institute of Pharmaceutical Education and Research, Kolkata, West Bengal 700054, India.

***Corresponding Author**

Dr. Subhadeep Roy
Assistant Professor
Department of Pharmacology and Toxicology
National Institute of Pharmaceutical Education and Research,
Kolkata, West Bengal, India, 700054.
Email id- subhadeeproy.good@gmail.com.

Table S1. Panel of selected breast cancer-associated markers used in this study.

**S.No**	**Marker**	**Cell Type**
1.	CD20	B Cell
2.	CD24	Cancer stem cells
3.	CD34	Hematopoietic stem cells (HSCs)
4.	CD45	Stem cells/ EMT marker
5.	CD73	Mesenchymal Stem Cell
6.	CD90	Basal-like subtype 2/Stem cell
7.	CD105	Mesenchymal Stem Cell/Stromal cell/ fibroblast
8.	E-cadherin	Endothelial cell/ EMT
9.	Laminin	Mesenchymal Cell
10.	Fibronectin	Fibroblast

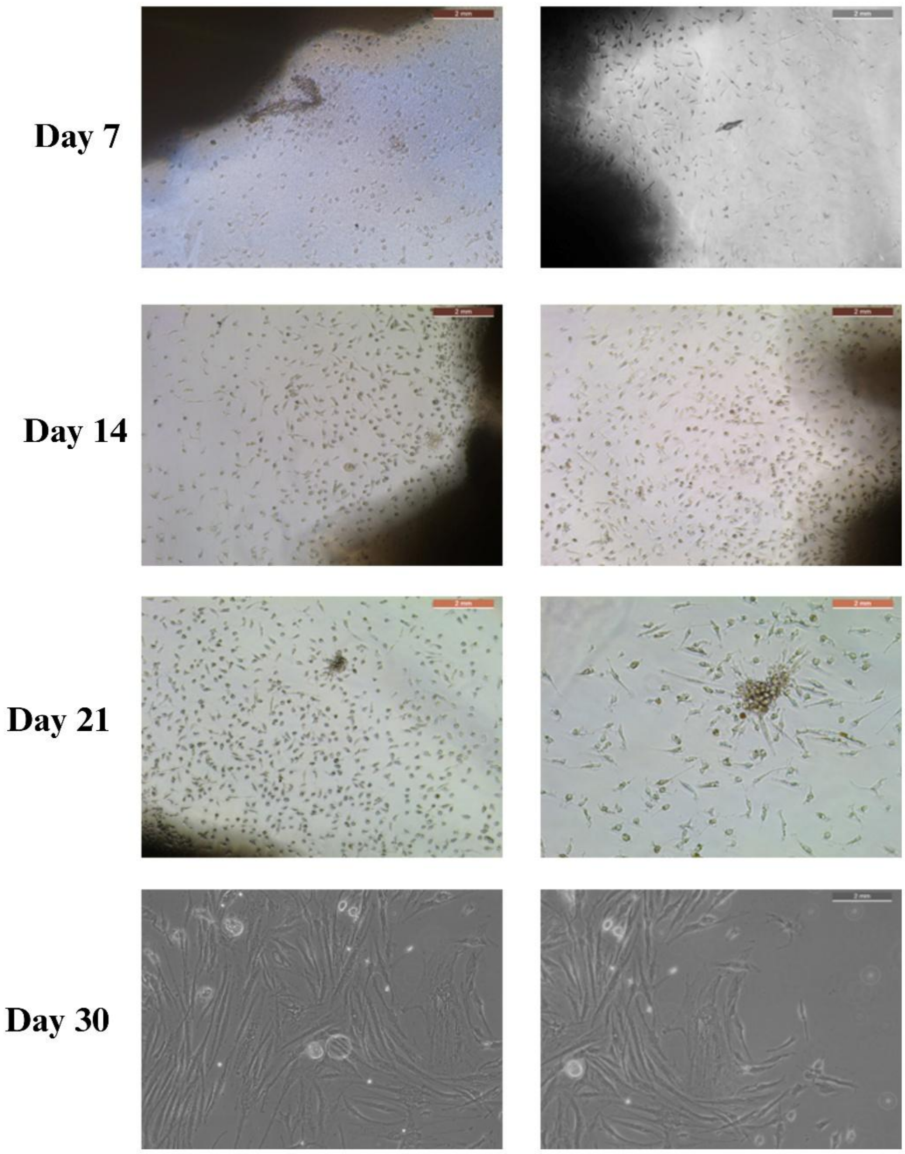

Figure S1. Progressive migration of heterogeneous cell populations within breast cancer PDOs observed on days 7, 14, 21, and 30. The time-course imaging highlights dynamic cellular organization and expansion, reflecting the structural and phenotypic complexity of the tumor microenvironment over the culture period.

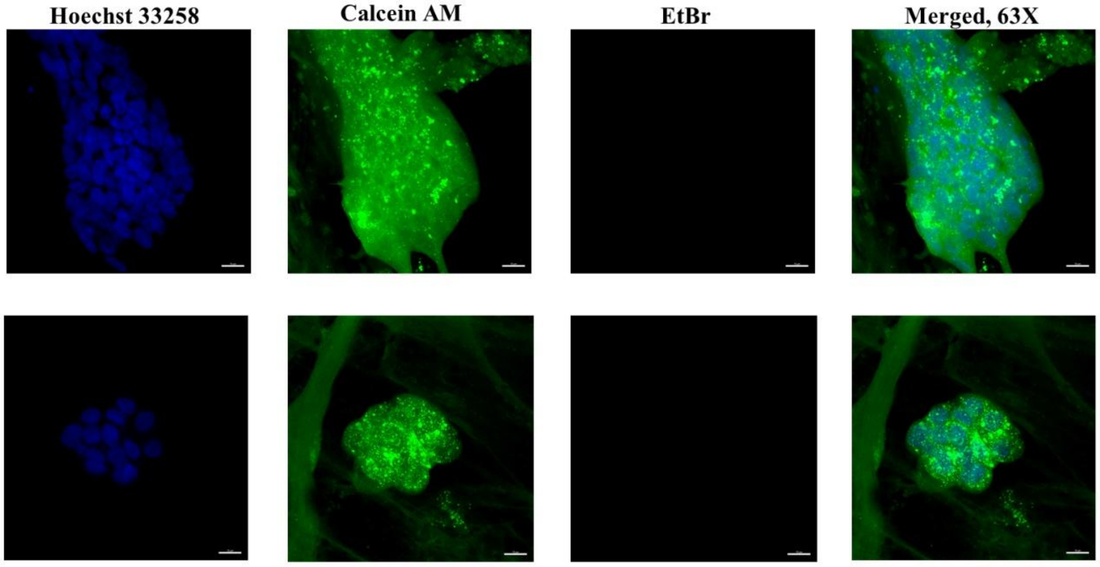

Figure S2. Unprocessed raw images from live/dead viability assays of breast cancer PDOs after 30 days of culture. The images depict cellular viability and spatial distribution of live (green) and dead (red) cells, reflecting organoid health and integrity at the endpoint.

INSTITUTIONAL ETHICS COMMITTEE

Chittaranjan National Cancer Institute
An autonomous body under the Ministry of Health & Family Welfare, Govt. Of India
37, S.P.Mukherjee Road, Kolkata-700026, WB, India
Tel: 2475-9313/8057, Fax: +91-33-2475-7606
Web: www.cnci.ac.in

Chairman
Prof. Tamal Kanti Ghosh
Professor, Dept. of Pathology, Deben Mahata Govt Medical College, Purulia

Member Secretary
Dr. Sutapa Mukherjee, CNCI, Kolkata

Members
Prof. (Dr.) Santanu Tripathi, Director, Centre for Clinical Research, John C Martin Centre for Liver Research and Innovations, Indian Institute of Liver and Digestive Sciences, Sonarpur, Kolkata
Dr. Syed Mohammad Naser, Professor, Pharmacology, Calcutta National Medical College, Kolkata
Prof. (Dr.) Susanta Roychoudhury, Indian Institute of Chemical Biology, IICB, Kolkata
Dr. Kalyan Kusum Mukherjee, Head, Medical Oncology, Head, Clinical & Translational Research, CNCI, Kolkata
Dr. Sankar Sengupta, Medical Superintendent, CNCI, Kolkata
Dr. Debarshi Lahiri, CNCI, Kolkata
Dr. Amitabha Ray, Dept of Radiotherapy, CNCI, Kolkata
Dr. Provas Sadhukhan, National Institute for Cholera and Enteric Disease (NICED), Kolkata
Dr. Sankhadeep Dutta, CNCI, Kolkata
Dr. Kartiki V. Desai, Professor, National Institute of Biomedical Genomics (NIBMG), Kalyani
Mr. Masud Karim High Court, Kolkata
Ms. Sutapa Biswas, CFI, Kolkata
Mrs. Susmita Banerjee

IEC Ref: CNCI-IEC-JC3-2024-103

Date: 11.03.2024

Dr. Jayanta Chakraborty
Director & Head, Dept. of Surgical Oncology

Sub.: IEC decision on review of the project submitted for approval.

Protocol Title: ***Development of Anticancer Drugs Using Primary Culture and Cancer Organoid Model.***

Study Site: Chittaranjan National Cancer Institute, Kolkata

Full Board Review Meeting of IEC was conducted in physical mode on **22nd February, 2024**. The Chairman and members of the committee reviewed the project and other related documents submitted by you for the proposed study entitled ***"Development of Anticancer Drugs Using Primary Culture and Cancer Organoid Model."***

The following documents were reviewed:

1. Project with Summary
2. Informed Consent Sheets
3. Administrative Approval
4. Academic/Scientific Committee approval

The committee should be informed:

I. About the progress of the study annually
II. Any changes in the protocol and patient information/ informed consent documents, prior to their implementation

The Project is approved by the IEC to develop Organoid Model only.

Final report of the study shall have to be submitted to the IEC in all cases, even when the study is abandoned for any reason(s).

Yours Sincerely,

Sutapa Mukherjee
Member Secretary
IEC, CNCI

Member Secretary
Institutional Ethics Committee
Chittaranjan National Cancer Institute
37, S. P. Mukherjee Road, Kokata-700026

Chairman
IEC, CNCI

Chairman
Institutional Ethics Committee
Chittaranjan National Cancer Institute
37, S. P. Mukherjee Road, Kokata-700026

PATIENT CONSENT FORM

Name of study: **Development of Anticancer Drugs Using Primary Culture and Cancer Orgonoid Model.**

Patient/hospital ID: CNCIN230000010611

I, Mr./Mrs./Miss [redacted] of ____________________, S/D/W ____________________, resident of ____________________, aged 43 yrs. do hereby declare that I am voluntarily giving my consent to participate in the study titled "**Development of Anticancer Drugs Using Primary Culture and Cancer Orgonoid Model.**"

I have been explained to my full satisfaction in my own language about the procedure involved in the study along with my right to refuse to participate in the study at any time during the course of the study. My refusal however is not going to affect my right to receive the treatment of my illness from the department.

I do hereby declare that I will provide medical history of the disease, undergo clinical examination & allow collection of necessary clinical material.

Name of the declarant [redacted]

Signature of the declarant [redacted]

Dated 22/01/2024

Place: Kolkata

Name of the witness [redacted]

Signature of the witness [redacted]

Dated 22/01/2024

Place: Kolkata

Name of the investigator ____________________

Signature of the investigator ____________________

Dated ____________________

Place: Kolkata

PATIENT CONSENT FORM

Name of study: **Development of Anticancer Drugs Using Primary Culture and Cancer Orgonoid Model.**

Patient/hospital ID: CNCINQ9000000 7024

I, Mr./Mrs./Miss [redacted], S/D/W of [redacted], resident of Sekhpurah, Mednipur, aged 62 yrs. do hereby declare that I am voluntarily giving my consent to participate in the study titled **"Development of Anticancer Drugs Using Primary Culture and Cancer Orgonoid Model."**

I have been explained to my full satisfaction in my own language about the procedure involved in the study along with my right to refuse to participate in the study at any time during the course of the study. My refusal however is not going to affect my right to receive the treatment of my illness from the department.

I do hereby declare that I will provide medical history of the disease, undergo clinical examination & allow collection of necessary clinical material.

Name of the declarant [redacted]

Signature of the declarant [redacted]

Dated 23/01/24

Place: Kolkata

Name of the witness [redacted]

Signature of the witness [redacted]

Dated 23/01/24

Place: Kolkata

Name of the investigator __________

Signature of the investigator __________

Dated__________

Place: Kolkata

PATIENT CONSENT FORM

Name of study: **Development of Anticancer Drugs Using Primary Culture and Cancer Orgonoid Model.**

Patient/hospital ID: CNCIN23000008886

I, Mr./Mrs./Miss [redacted], S/D/W of __________, resident of Pratapgarh, Dumdum, aged 63 yrs. do hereby declare that I am voluntarily giving my consent to participate in the study titled **"Development of Anticancer Drugs Using Primary Culture and Cancer Orgonoid Model."**

I have been explained to my full satisfaction in my own language about the procedure involved in the study along with my right to refuse to participate in the study at any time during the course of the study. My refusal however is not going to affect my right to receive the treatment of my illness from the department.

I do hereby declare that I will provide medical history of the disease, undergo clinical examination & allow collection of necessary clinical material.

Name of the declarant [redacted]

Signature of the declarant [redacted]

Dated 16/04/2024

Place: Kolkata

Name of the witness [redacted]

Signature of the witness [redacted]

Dated 16/04/2024

Place: Kolkata

Name of the investigator __________

Signature of the investigator __________

Dated __________

Place: Kolkata

PATIENT CONSENT FORM

Name of study: **Development of Anticancer Drugs Using Primary Culture and Cancer Orgonoid Model.**

Patient/hospital ID: CNCIN0000010425

I, Mr./Mrs./Miss [redacted], S/D/W of [redacted], resident of Kanya Nagar Colony, South 24 Pgs, aged 41 yrs. do hereby declare that I am voluntarily giving my consent to participate in the study titled "**Development of Anticancer Drugs Using Primary Culture and Cancer Orgonoid Model.**"

I have been explained to my full satisfaction in my own language about the procedure involved in the study along with my right to refuse to participate in the study at any time during the course of the study. My refusal however is not going to affect my right to receive the treatment of my illness from the department.

I do hereby declare that I will provide medical history of the disease, undergo clinical examination & allow collection of necessary clinical material.

Name of the declarant [redacted]

Signature of the declarant [redacted]

Dated 20/12/2023

Place: Kolkata

Name of the witness [redacted]

Signature of the witness [redacted]

Dated 20/12/2023

Place: Kolkata

Name of the investigator ____________

Signature of the investigator ____________

Dated ____________

Place: Kolkata

PATIENT CONSENT FORM

Name of study: **Development of Anticancer Drugs Using Primary Culture and Cancer Orgonoid Model.**

Patient/hospital ID:

I, Mr./Mrs./Miss [redacted], S/D/W of ______________, resident of Barasiyakur, Santipur, Nadia, west bengal, aged 63 yrs. do hereby declare that I am voluntarily giving my consent to participate in the study titled **"Development of Anticancer Drugs Using Primary Culture and Cancer Orgonoid Model."**

I have been explained to my full satisfaction in my own language about the procedure involved in the study along with my right to refuse to participate in the study at any time during the course of the study. My refusal however is not going to affect my right to receive the treatment of my illness from the department.

I do hereby declare that I will provide medical history of the disease, undergo clinical examination & allow collection of necessary clinical material.

Name of the declarant [redacted]

Signature of the declarant [redacted]

Dated 3/10/24

Place: Kolkata

Name of the witness [redacted]

Signature of the witness [redacted]

Dated 3/10/24

Place: Kolkata

Name of the investigator ______________

Signature of the investigator ______________

Dated ______________

Place: Kolkata

PATIENT CONSENT FORM

Name of study: **Development of Anticancer Drugs Using Primary Culture and Cancer Orgonoid Model.**

Patient/hospital ID: CNCIN24000000 2142

I, Mr./Mrs./Miss [redacted], S/D/W of____________________, resident of ____________________, aged 52 yrs. do hereby declare that I am voluntarily giving my consent to participate in the study titled **"Development of Anticancer Drugs Using Primary Culture and Cancer Orgonoid Model."**

I have been explained to my full satisfaction in my own language about the procedure involved in the study along with my right to refuse to participate in the study at any time during the course of the study. My refusal however is not going to affect my right to receive the treatment of my illness from the department.

I do hereby declare that I will provide medical history of the disease, undergo clinical examination & allow collection of necessary clinical material.

Name of the declarant [redacted]

Signature of the declarant [redacted]

Dated 16/04/2024

Place: Kolkata

Name of the witness [redacted]

Signature of the witness [redacted]

Dated 16/04/24

Place: Kolkata

Name of the investigator ____________________

Signature of the investigator ____________________

Dated____________________

Place: Kolkata
